# Nectin-4 expression contributes to tumor proliferation, angiogenesis and patient prognosis in human pancreatic cancer

**DOI:** 10.1186/s13046-015-0144-7

**Published:** 2015-03-28

**Authors:** Satoshi Nishiwada, Masayuki Sho, Satoshi Yasuda, Keiji Shimada, Ichiro Yamato, Takahiro Akahori, Shoichi Kinoshita, Minako Nagai, Noboru Konishi, Yoshiyuki Nakajima

**Affiliations:** Department of Surgery, Nara Medical University, 840 Shijo-cho, Kashihara, Nara 634-8522 Japan; Department of Pathology, Nara Medical University, 840 Shijo-cho, Kashihara, Nara 634-8522 Japan

**Keywords:** Nectin-4, Prognostic factor, Pancreatic cancer, Tumor angiogenesis

## Abstract

**Background:**

Nectin-4 belongs to the nectin family that has diverse physiological and pathological functions in humans. Recent studies have also suggested some roles for Nectin-4 in several human cancers. However, the precise roles and clinical relevance of Nectin-4 in tumors are largely unknown.

**Methods:**

Nectin-4 expression was investigated in 123 patients with pancreatic cancer by immunohistochemistry. Furthermore, we investigated the association of Nectin-4 in pancreatic cancer with tumor proliferation, angiogenesis and immunity by using immunohistochemistry and siRNA interference method.

**Results:**

Patients with high Nectin-4 expression had poorer postoperative prognosis than those with low expression. Importantly, multivariate analysis indicated that Nectin-4 expression had a significant independent prognostic value in pancreatic cancer (*HR* = 1.721, 1.085-2.730; *P* = 0.021). Tumor Nectin-4 expression was significantly correlated with Ki67 expression. In addition, siRNA-mediated gene silencing of Nectin-4 significantly inhibited the cell proliferation in human pancreatic cancer cells, Capan-2 and BxPC-3. Furthermore, Nectin-4 expression was also positively correlated with VEGF expression and intratumoral microvessel density. However, there were no significant correlations of tumor Nectin-4 expression with tumor-infiltrating T cells.

**Conclusion:**

Nectin-4 is a significant prognostic predictor, and may play a critical role in pancreatic cancer. Nectin-4 may be novel therapeutic target for pancreatic cancer.

## Background

Pancreatic cancer is highly aggressive and is the fourth leading cause of cancer deaths in the Unite States, and the number of patients has been increasing [1]. Although several advancements in the treatment including new reagents and regimens of chemotherapy and radiotherapy have recently been introduced, the prognosis of patients remains poor [2-7]. Although curative surgical resection has been considered to be the only chance for cure or long-term survival, more than half of the patients already have distant metastases at the time of initial evaluation [8]. Therefore, the surgery is indicated for only 20-30% of patients [6]. Thus, the identification of novel targets and development of new therapeutic approaches are required to improve patient prognosis [9-11].

The nectins (Nectin-1, Nectin-2, Nectin-3, and Nectin-4) and nectin-like molecules (Necl) are families of Ca2 + -independent immunoglobulin-like cell adhesion molecules. These members are important in the formation and maintenance of adherens junctions and tight junctions [12-14]. Furthermore, each member has also diverse functions and acts independently or interactively. Although Nectin-1, 2, and 3 are widely expressed in normal adult tissues, Nectin-4, also known as PVRL4 (poliovirus-receptor-like 4), is expressed specifically in the embryo and placenta [15,16]. It was recently reported that Nectin-4 is overexpressed in several human cancers, including lung, ovarian, and breast cancer [17-20]. It was also demonstrated that a soluble form of Nectin-4 has a potential as a diagnostic marker for several cancers [17-20]. Furthermore, a few clinical studies have shown that there were significant inverse correlations between tumor Nectin-4 expression and the prognosis of the patients with lung and breast cancers [17,19].

Besides these clinical studies, several recent basic studies have revealed that the potential roles and functions of Nectin-4 in tumor biology [15,16,19,21]. One study has shown that Nectin-4 contributes to the cell growth and proliferation of tumors through the Rac1-signaling pathway in human lung adenocarcinoma cells [19]. In other study, Nectin-4 promotes the attachment of individual cells to each other on a juxtaposed cell, and maintains the transformed properties of breast cancer cells *in vitro*. In addition, the knockdown of Nectin-4 by transfection of siRNA or the intravenous injections of anti-Nectin-4 monoclonal antibody inhibits tumor growth and reduces cell-cell contact in breast cancer cell lines *in vivo* [21]. Furthermore, Nectin-4 may have unknown functions, since other nectins and Necls are reported to play important roles in various conditions including acquired immunity and angiogenesis [22-27].

Although the accumulating evidence indicates that Nectin-4 may play a critical role in tumor biology, the precise roles of Nectin-4 in tumor progression and metastasis in human cancers are not fully elucidated. Furthermore, to our knowledge, its clinical significance in pancreatic cancer has not been addressed. In this study, we tried to clarify the clinical importance of Nectin-4 expression in human pancreatic cancer. In addition, based on the previous studies on Nectin-4, we aimed to better understand the underlying functions of Nectin-4 in pancreatic cancer.

## Methods

### Patients

We examined 123 pancreatic cancer patients who had undergone surgery at Nara Medical University Hospital between 1992 and 2008. The median age of the patients was 66 years, with a range of 33 to 82 years. No one received preoperative anticancer treatment. All tumors were diagnosed as pancreatic ductal adenocarcinoma. Tissues, both cancerous and non-cancerous, were obtained from resected specimens and were then rapidly frozen at -80°C for storage until use. The remainder of each specimen was fixed in 10% phosphate-buffered formalin and embedded in paraffin. Tumors were classified according to the TNM staging system of the International Union Against Cancer (UICC) [28]. Follow-up was until death or January 2014. Written informed consent was obtained from all patients before treatment, according to our institutional guidelines. This study was approved by the institutional review board (Nara Medical University Ethics Committee).

### Cell lines and culture

The human pancreatic cancer cell lines, Capan-2 and BxPC-3 were obtained from RIKEN BioResource Center and cultured in RPMI 1640 supplemented with 10% heat-inactivated fetal bovine serum (FBS).

### Immunohistochemistry

The paraffin embedded full sections were stained using a DAKO EnVision system (DAKO Cytomation, Japan), according to the manual provided by the manufacturer. As primary antibodies, the anti-human Nectin-4 antibody (AF2659, 1:40 dilution; R&D Systems, USA) was used. The anti-human CD4, CD8, CD45RO, CD31, and Ki67 antibodies (DAKO) were also used. Formalin-fixed, paraffin-embedded tissues were cut into 5-μm sections, deparaffinized, and rehydrated in a graded series of ethanol. Antigen retrieval was done by heating tissue sections using a Target Retrieval Solution, pH 9.0 (DAKO). To block endogenous peroxidase, sections were immersed in 0.3% solution of hydrogen peroxide in absolute methanol for 5 minutes at room temperature and washed in fresh PBS for 3 times, each of 5 minutes duration. Purified each mAb was added and incubated overnight at 4°C. Sections were washed in PBS for 3 times, each of 5 minutes duration, and then we use EnVision+, Mouse/HRP or Rabbit/HRP (DAKO) according to the instructions of the manufacturer. Sections were counterstained with hematoxylin, dehydrated in ethanol, cleared in xylene, and coverslipped.

### Evaluation of immunostaining

Immunohistochemistry for Nectin-4, at least 200 tumor cells were scored per field at × 400 magnification. The positive cell was defined as the cells with strongly and clearly brown immunostained cytoplasm. Specimens with a ≥50% Nectin-4 positive tumor cells were classified as Nectin-4 high status, and others were as Nectin-4 low. Since staining was constantly intense in most cases, the intensity of each sample was not counted in this study. Immunohistochemistry for CD4+, CD8+, and CD45RO+ T cells was evaluated. An average number of >50 accumulating CD4+, CD8+, and CD45RO + tumor-infiltrating T lymphocytes (TILs) per field at × 200 magnification were scored in five fields. For the microvessel counting, the five most highly vascularized areas were counted at × 200 magnification, and the average counts were recorded [29,30]. The mean microvessel count of these tumors was 55.9. To evaluate the Ki67 expression, at least 1,000 tumor cells were scored in the invasive front of tumors at a magnification of × 400, and the percentage of tumor cells showing positive staining was calculated [31]. We classified into two groups according to the median of positivity of Ki67 expression. Authorized two pathologists who had no knowledge of the patients’ clinical status and outcome evaluated immunohistochemistry. In case of disagreement, the slides were re-evaluated until agreement was reached.

### Extraction of total RNAs and real-time reverse transcriptase polymerase chain reaction (PCR) analysis

Total RNA was isolated from resected frozen specimens by using RNAspin Mini (GE Healthcare, Tokyo, Japan) and the first-strand cDNA was synthesized from 1 μg RNA using a high-capacity cDNA reverse transcription kit (Applied Biosystems, USA), according to the manufacturer’s protocol. Real-time quantitative PCR analysis was carried out using an ABI Prism 7700 sequence detector system (Applied Biosystems). All primer/probe sets were purchased from Applied Biosystems. PCR was carried out using the TaqMan Universal PCR Master Mix (Applied Biosystems) using 1 μl of cDNA in a 20 μl final reaction volume. The PCR thermal cycle conditions were as follows: initial step at 95°C for 10 min, followed by 40 cycles of 95°C for 15 seconds and 60°C for 1 min. The expression level of the housekeeping gene β2-microglobulin was measured as an internal reference with a standard curve to determine the integrity of template RNA for all specimens. The ratio of the mRNA level of each gene was calculated as follows: (absolute copy number of each gene)/(absolute copy number of β2-microglobulin).

### Small interfering RNA (siRNA) transfection of Nectin-4

For our transfection analyses, Capan-2 and BxPC-3 cells were seeded in 6-well plates, and transfected either with control RNA (Santa Cruz Biotechnology, USA) or with 10 nM of siRNA of Nectin-4. Transfections were carried out using the Lipofectamine system (Invitrogen) in accordance with the manufacturer’s protocol when cells ware achieved about 30% confluent. The Nectin-4 siRNA duplexes, generated with 30-dTdT overhangs and prepared by QIAGEN (USA), were chosen against the DNA target sequences as follows: (Nectin-4 target sequence: 5′-CAGAGCAGTATTAATGATGCA-3′).

### Cell viability assay

Cell viability was determined using the Cell-titer 96 aqueous one solution cell proliferation assay kit, according to the instruction manual (Promega Corporation, USA). Briefly, aliquots of 1 × 10^3^ cells per well were cultured in 96-well plates with Nectin-4 or control siRNA for 72 hours. After incubation, MTS [3-(4,5-dimethyl-2-yl)-5-(3-carboxy-methoxyphenyl)-2-(4-sulfophenyl)-2H-tetrazolium, inner salt] reagent (Promega) was added to each well and incubated for an additional 1 hour. The absorbance at 492 nm was recorded with a 96-well plate reader. Each experiment was performed in triplicate and repeated at least thrice.

### Preparation of cell lysates and Western blot analysis

We resolved the cell lysates in SDS-polyacrylamide gels and transferred them onto polyvinylidene difluoride membranes (Millipore, Ltd. USA), which were then blocked in 5% skim milk at room temperature for 1 hour. The membranes were incubated with the indicated the anti-human PVRL4 antibody (ab192033, 1:1000 dilution; Abcam, UK) overnight at 4°C, and then incubated with horseradish peroxidase-conjugated IgG (Santa Cruz Biotechnology). We detected peroxidase activity on X-ray films using an enhanced chemiluminescence detection system.

### Statistical analysis

The overall survival time was calculated from the date of surgery to the date of death. Kaplan–Meier survival calculations and the corresponding log-rank tests were carried out to determine differences in survival rates. Multivariate analysis was done using the Cox regression model. We used the term of residual tumor status as R factor, tumor status as T factor, nodal status as N factor, and metastatic status as M factor in tumor-node-metastasis classification, respectively. The chi-square test or Fisher’s exact test was used to analyze the significance of the association between the expression of Nectin-4 and clinicopathological factors. Other data were analyzed using the Student’s *t* test or the Mann-Whitney *U* test as appropriate to determine significant differences. The Spearman’s rank test was also used to determine the correlation between two variables. A *P* value <0.05 was considered statistically significant. The statistical analyses were performed using the SPSS^®^ software program, version 19.0 (SPSS, Chicago, IL, USA).

## Results

### Nectin-4 expression and prognostic value in human pancreatic cancer

**Figure 1:**
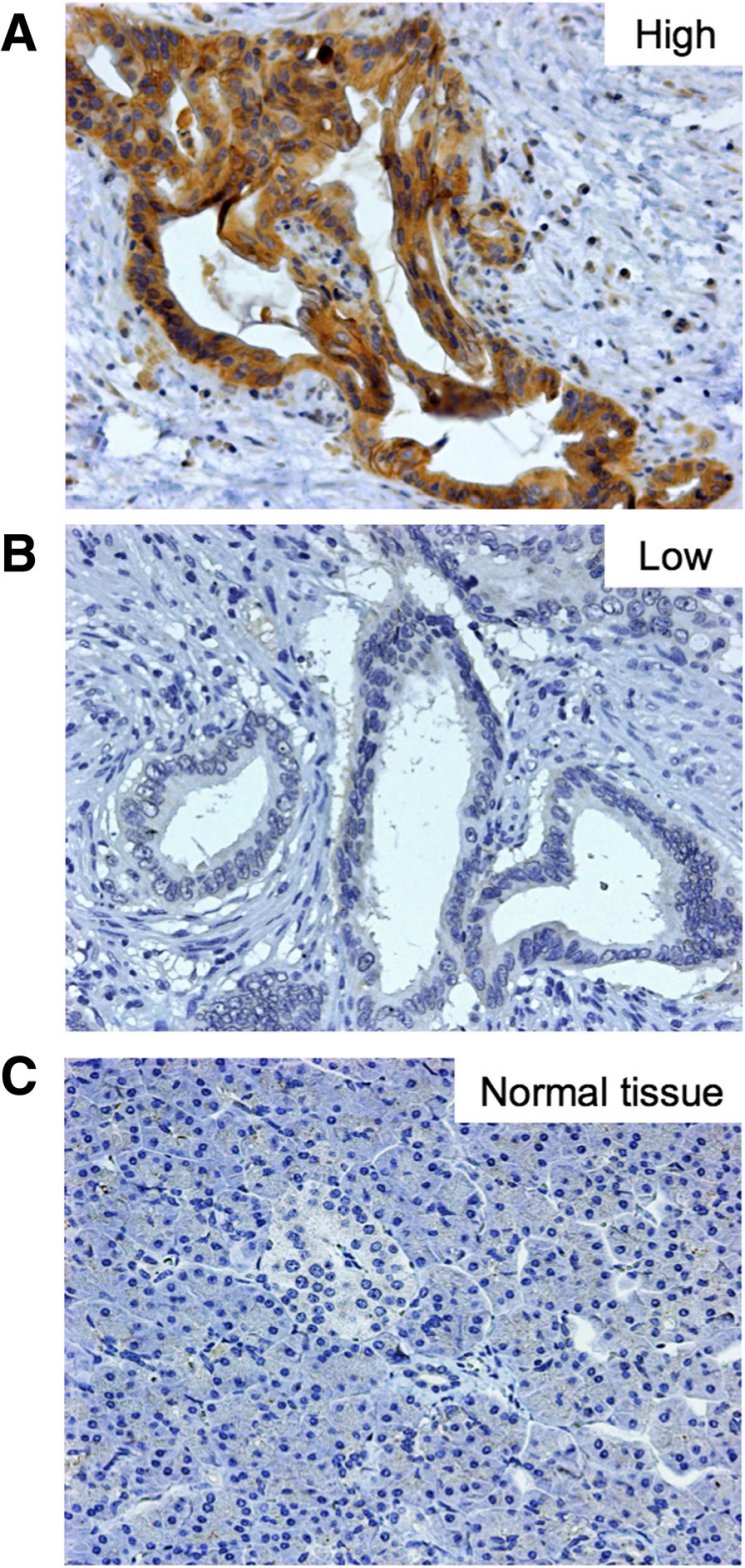
**Nectin-4 expression in human pancreatic cancer tissues. (A**,**B)** Tumor cell with high or low Nectin-4 expression. **(C)** There was limited expression of Nectin-4 in non-cancer tissues including islet cells, acinar cells and normal gland. Representative case of tumor Nectin-4 expression. Original magnification, ×200.

**Figure 2:**
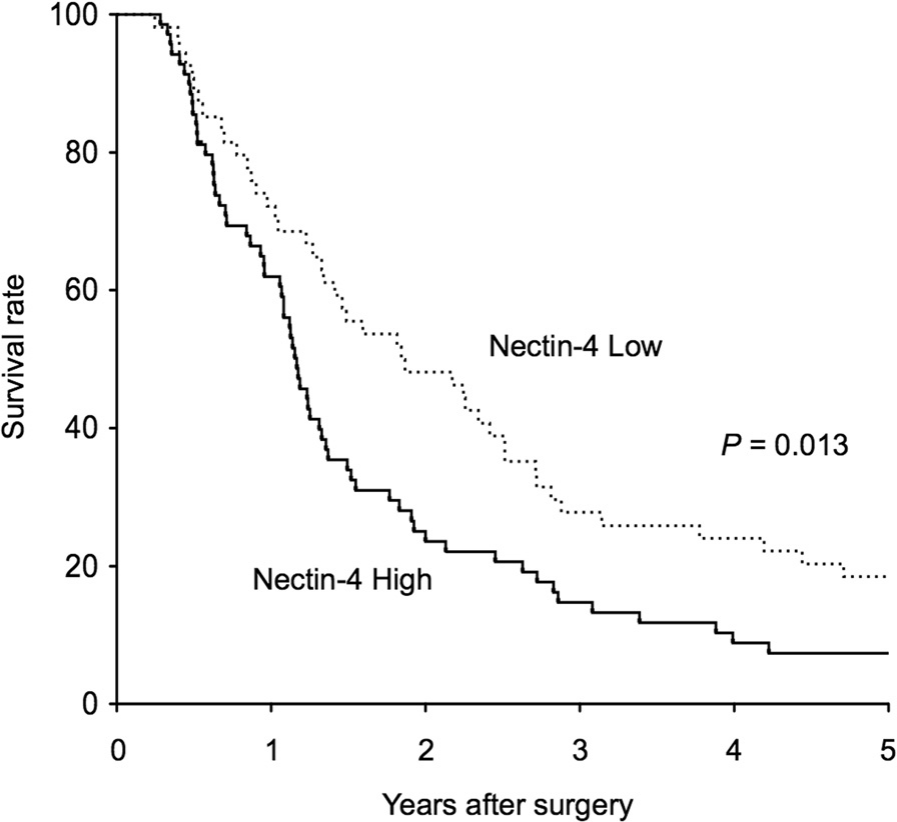
**Prognostic value of Nectin-4 status in human pancreatic cancer.** Overall survival of 123 patients with pancreatic cancer according to tumor Nectin-4 status. Patients with tumors of high Nectin-4 expression had significantly poorer prognosis than those with the low expression (*P* = 0.013).

We first evaluated the Nectin-4 expression in 123 actual human pancreatic cancer tissues by immunohistochemistry. Nectin-4 was abundant and expressed mainly in the plasma membrane and cytoplasm of cancer cells (Figure 1A). On the other hand, there was limited expression of Nectin-4 in non-cancer tissues including islet cells, acinar cells and normal gland (Figure 1C). Overall, the mean percentage of Nectin-4 positive cells in pancreatic cancer tissues was 51.4% (standard deviation 23.5%). Each sample was classified into two groups according to more or less than 50% of positivity of Nectin-4 expression (Figure 1A,B). As a result, 69 (56.1%) patients were classified as high, while 54 (43.9%) patients were classified as low. Interestingly, the patients with Nectin-4 high tumor had significantly poorer postoperative prognosis than patients with Nectin-4 low (*P* = 0.013, Log-rank test; Figure 2). The median survival times were 426 days in the patients with Nectin-4 high and 682 days in Nectin-4 low. Postoperative recurrence was observed in 94.2% of Nectin-4 high group and 87.0% of low group at the time of analysis. The recurrence pattern in Nectin-4 high group was hematogenous in 31 (44.9%), local in 33 (47.8%), and peritoneal in 19 (27.5%). In Nectin-4 low group, those were 16 (29.6%), 25 (46.3%), and 17 (31.5%), respectively. There was no significant difference in recurrence pattern between two groups.

Next, we evaluated the correlations of the Nectin-4 expression with various clinicopathological factors. There were no significant differences between two groups in any clinicopathological findings including TNM status and pathological UICC stage (Table 1).

**Table 1:** **Comparison of clinicopathological characteristics according tumor Nectin-4 expression**

**Characteristics**		**Nectin-4 expression**		***P-*** **value** ^**a**^
	**Total n=123**	**low n=54 (%)**	**high n=69 (%)**	
Gender				
male	72	30 (55.6)	42 (60.9)	0.584
female	51	24 (44.4)	27 (39.1)	
Age, year				
< 66	59	27 (50.0)	32 (46.4)	0.690
>66	64	27 (50.0)	37 (53.6)	
Histopathological grading				
G1	34	13 (24.1)	21 (30.4)	0.873
G2	71	33 (61.1)	38 (55.1)	
G3	14	6 (11.1)	8 (11.6)	
G4	4	2 (3.7)	2 (2.9)	
Tumor status				
T1	8	4 (7.4)	4 (5.8)	0.299
T2	21	13 (24.1)	8 (11.6)	
T3	84	33 (61.1)	51 (73.9)	
T4	10	4 (7.4)	6 (8.7)	
Nodal status				
N0	50	24 (44.4)	26 (37.7)	0.466
N1	73	30 (55.6)	43 (62.3)	
Metastatic status				
M0	112	49 (90.7)	63 (91.3)	>0.999^b^
M1	11	5 (9.3)	6 (8.7)	
UICC stage				
IA, IB	21	13 (24.1)	8 (11.6)	0.358
IIA	26	10 (18.5)	16 (23.2)	
IIB	60	25 (46.3)	35 (507)	
III	5	1 (1.8)	4 (5.8)	
IV	11	5 (9.3)	6 (8.7)	

^a^Chi-square test, ^b^Fisher’s exact test.

*UICC* International Union Against Cancer.

### Correlation between the Nectin-4 and Ki67 expression in human pancreatic cancer

**Figure 3:**
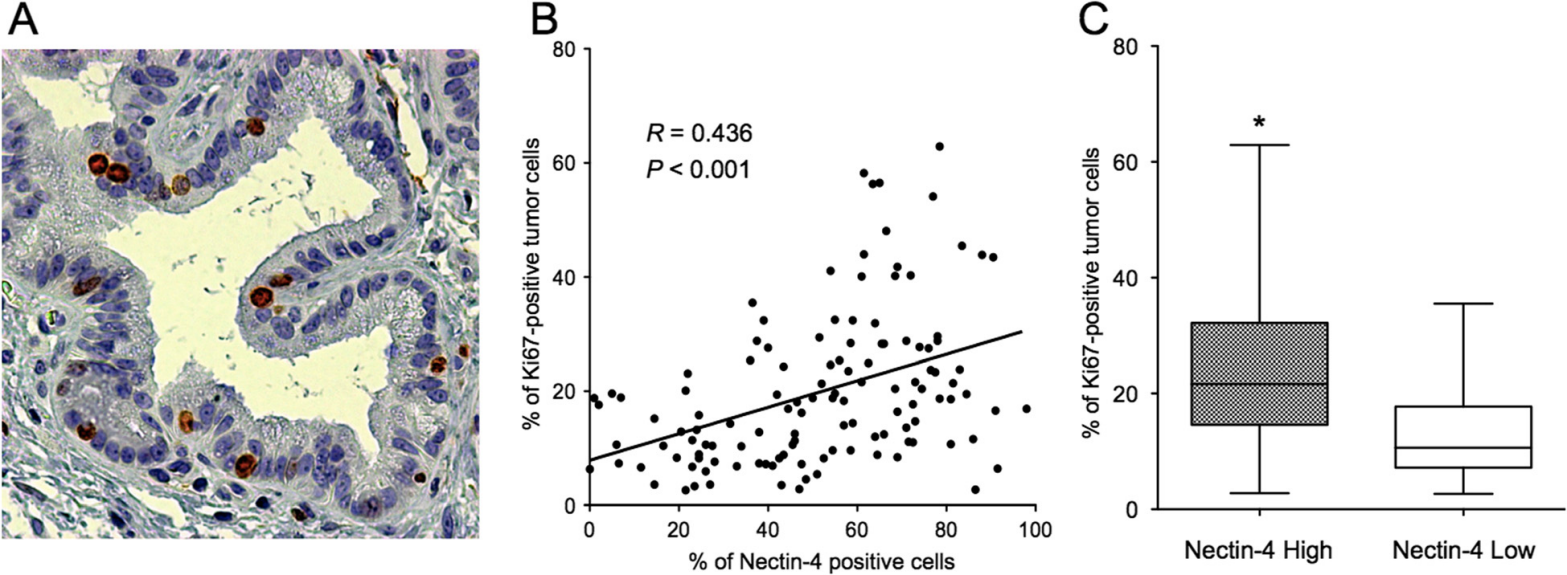
**Correlation between the Nectin-4 and Ki67 expression in human pancreatic cancer. (A)** Representative case of Ki67 expression. Original magnification, ×200. **(B)** There was a significant correlation between the percent of positive Nectin-4 and Ki67 expression levels (*R* = 0.436, *P* < 0.001, Spearman’s rank test). **(C)** The tumors with high Nectin-4 status had a significantly higher percentage of Ki67-positive tumor cells than the tumors with low Nectin-4 status (*P <0.001, Mann-Whitney *U* test).

Next, to further investigate the functions of Nectin-4 in pancreatic cancer, we evaluated the involvement of Nectin-4 in pancreatic cancer cell proliferation. We analyzed the correlation of Nectin-4 with Ki67, a proliferation marker, by immunohistochemistry (Figure 3A). Overall, the mean percentage of Ki67-positive tumor cells was 19.8%. The percent of Nectin-4 expression level was significantly correlated with the Ki67 expression level (*R* = 0.436, *P* < 0.001; Figure 3B). Furthermore, the Ki67-positive rate was significantly higher in the tumors with Nectin-4 high status than in tumors with Nectin-4 low (25.4 ± 1.7 vs. 12.7 ± 1.1%, *P* < 0.001; Figure 3C). These data suggested that Nectin-4 might contribute to tumor proliferation in human pancreatic cancer.

### Univariate and multivariate prognostic analysis of patients with pancreatic cancer

In the univariate analysis of the overall survival, high Nectin-4 expression was significant prognostic factor (*HR* =1.628, *P* = 0.014). For prognostic analysis, patients were categorized by the median age. The tumor status and histopathological grading were categorized according to the prognostic value determined by preliminary analysis. As a result, the other factors that significantly correlated with the patients’ overall survival were the tumor status, nodal status, metastatic status, residual tumor status, and Ki67 status. Furthermore, multivariate analysis indicated that tumor Nectin-4 status was a significant independent prognostic factor (*HR* = 1.721, *P* = 0.021) (Table 2). In addition, metastatic status and residual tumor status were defined as independent prognostic factors (Table 2). Taken together, these data suggested that Nectin-4 expression might play an important role in pancreatic cancer.

**Table 2:** **Univariate and multivariate prognostic analysis of 123 patients with pancreatic cancer**

**Characteristics**	**Univariate analysis**			**Multivariate analysis**		
	**HR**	**95% Cl**	**P-value** ^**a**^	**HR**	**95% Cl**	**P-value** ^**a**^
Age (66 vs. <66)	0977	0.672-1422	0905	1007	0.681-1,490	0.973
Gender (Female vs. Male)	1001	0.683-1468	0996	1021	0.691-1508	0.918
Tumor Status (T3-4vs. T1-2)	1.735	1.106-2.720	0.016^b^	1.136	0.649-1.990	0.655
Nodal Status (N1 vs. N0)	1.837	1.240-2.720	0.002^b^	1.387	0.877-2.193	0.162
Metastatic status (M1 vs. M0)	4450	2323-8525	<0.001^b^	2.713	1.352-5.446	0.005^b^
Residual Tumor (R1-2 vs. R0)	2.014	1.372-2958	<0.001^b^	1.811	1179-2.781	0.007^b^
Histopathological grading (G3-4 vs. G1-2)	1.361	0.810-2 285	0245	1 606	909-2 839	0.103
Ki67 (High vs. Low)	1462	1.004-2 129	0.048^b^	1 096	697-1.721	0.692
Nectin-4 status (High vs. Low)	1.628	1.105-2.398	0.014^b^	1.721	1.085-2.730	0.021^b^

^a^Cox regression model.

^b^Significant difference.

*HR*, hazard ratio; *CI*, confidence interval.

### Silencing of Nectin-4 inhibits proliferation of human pancreatic cancer cell

**Figure 4:**
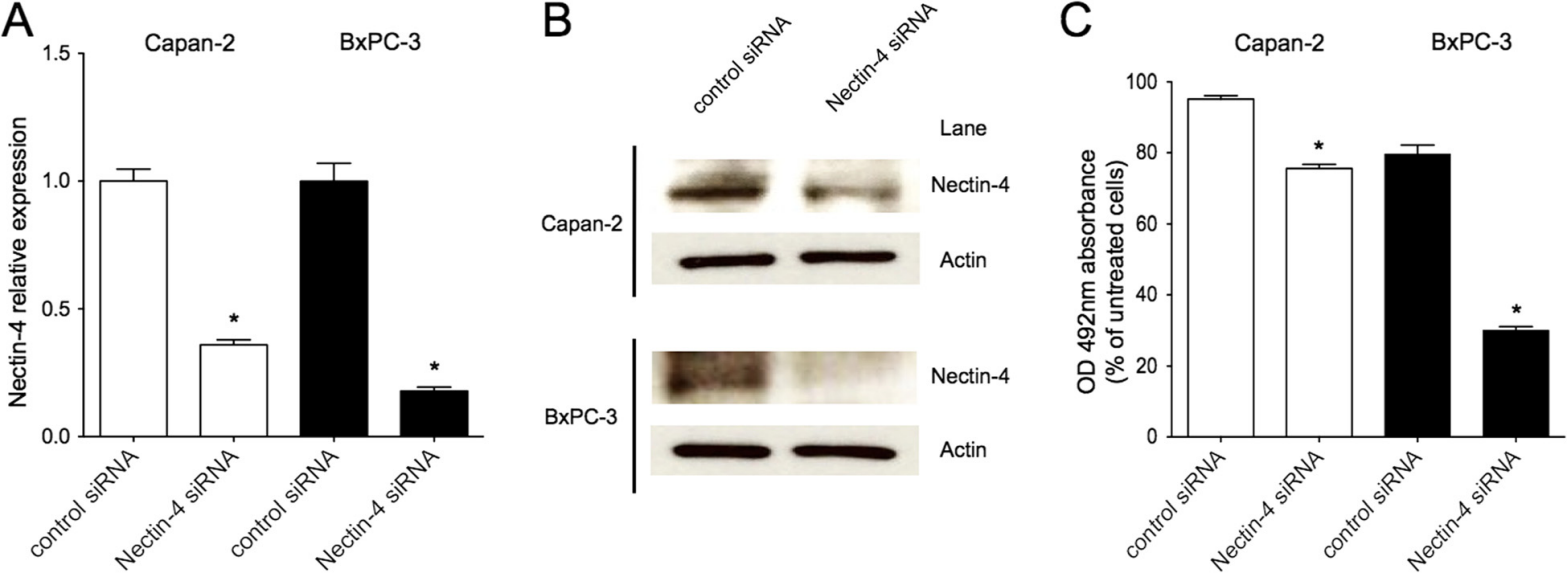
**Inhibition of Nectin-4 expression by gene silencing decreases cell proliferation in pancreatic cancer cells. (A**,**B)** Capan-2 and BxPC-3 cells were transfected with Nectin-4 siRNA or control RNA. The relative expression of Nectin-4 was significantly reduced in both cells when transfected with Nectin-4 siRNA for up to 72 hours as determined by quantitative real-time PCR and Western blot analysis (n = 4 of each group). **(C)** Cell proliferation was significantly inhibited by Nectin-4 gene silencing in both cells after 72 hours incubation as determined by MTS assay (n = 6 of each group). *P < 0.001 versus control siRNA (Student’s *t* test).

Furthermore, we directly examined the function of Nectin-4 in pancreatic cancer cell using siRNA silencing. When transfected with Nectin-4 siRNA in human pancreatic cancer cell lines, Capan-2 and BxPC-3, for up to 72 hours, both mRNA and protein expressions of Nectin-4 were substantially reduced (Figure 4A,B). As a result, cell proliferation was significantly suppressed by Nectin-4 gene silencing in these cells (Figure 4C). Data indicated that Nectin-4 might be critically involved in the proliferation of Capan-2 and BxPC-3 cells.

### Association of Nectin-4 with vascular endothelial growth factor (VEGF) expression and angiogenesis

**Figure 5:**
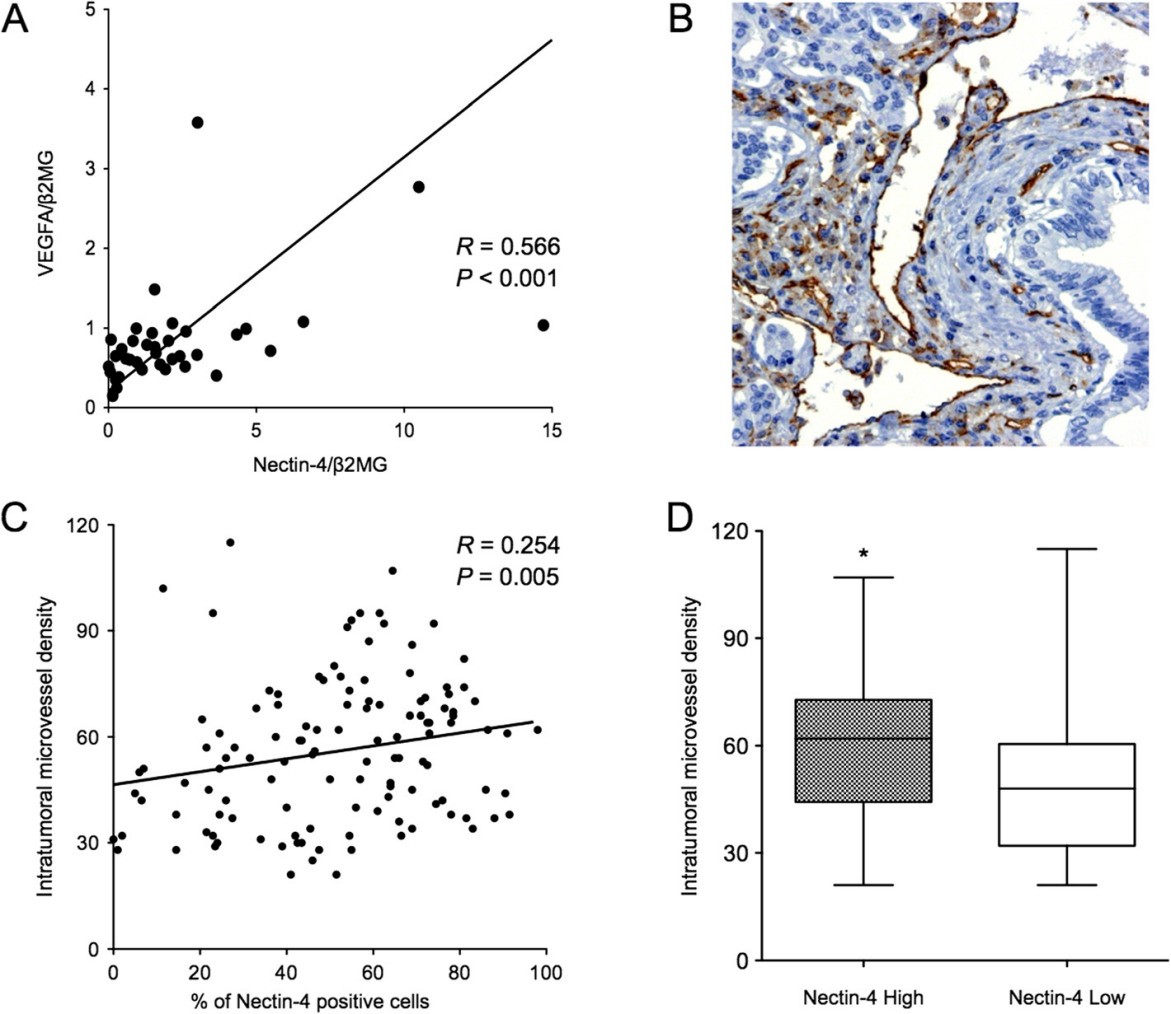
**Relationships between Nectin-4 expression and angiogenesis in human pancreatic cancer. (A)** Positive correlation between Nectin-4 and VEGF expression in human pancreatic cancer by quantitative real-time PCR (n = 38, *R* = 0.566, *P* < 0.001, Spearman’s rank test). **(B)** Representative immunohistochemical staining of CD31 in human pancreatic cancer tissues. Original magnification × 200. **(C)** There was a significant correlation between the percent of positive Nectin-4 and intratumoral microvessel density (*R* = 0.254, *P* = 0.005, Spearman’s rank test). **(D)** The intratumoral microvessel density in the tumors with high Nectin-4 status was significantly higher than that in the tumors with low Nectin-4 status (*P =0.001, Mann-Whitney *U* test).

We then analyzed the relationship between Nectin-4 and angiogenesis. There was a significant positive correlation between Nectin-4 and VEGF expressions determined by real-time PCR analysis (*R* = 0.566, *P* < 0.001; Figure 5A). We also examined the intratumoral microvessel density (IMD) by immunohistochemistry (Figure 5B). The microvessel count ranged from 20.7 to 115.0, with the mean count of 55.9. The percent of Nectin-4 expression level was significantly correlated with the IMD (*R* = 0.254, *P* = 0.005; Figure 5C). Furthermore, there was also a significant correlation between Nectin-4 status and IMD (*P* = 0.001; Figure 5D). These data suggested that Nectin-4 might play important role in angiogenesis in human pancreatic cancer.

### Association between Nectin-4 expression and tumor infiltrating lymphocytes

Finally, we investigated the immunomodulatory function of Nectin-4 in human pancreatic cancer. We performed immunohistochemical analysis of TILs including CD4, CD8, and CD45RO. The mean numbers of CD4+, CD8+, and CD45RO+ T cells counts were 165.0, 199.5, and 205.9, respectively. As a result, there were no significant correlations of tumor Nectin-4 expression with TILs in any T cell subsets (data not shown).

## Discussion

Accumulating evidence indicates that the nectins and Necls belonging to the nectin family have diverse physiological and pathological functions in humans [12-14]. Nectins have been originally investigated in cell adhesion, and play critical roles in various physiological conditions including tissue development. Besides physiological roles, nectins and Necls have also been shown to play important roles in tumor biology [32,33]. In particular, there are only limited studies on Nectin-4 in tumor biology and clinical cancers [17-20]. This study focused on the clinical significance of Nectin-4 expression in pancreatic cancer. We first examined Nectin-4 expression in actual pancreatic cancer tissue by immunohistochemistry. As a result, the intense expression was found in most human pancreatic cancer tissues, while limited expression was observed in non-cancer tissues of the pancreas. Interestingly, patients with high Nectin-4 expression had significantly poor prognosis in comparison with patients with low Nectin-4 expression. More importantly, the multivariate analysis demonstrated that tumor Nectin-4 status was defined as a significant independent prognostic factor for pancreatic cancer patients. A few previous reports have shown that Nectin-4 expression contributes to the postoperative prognosis of patients with malignant tumors including lung adenocarcinoma and breast cancer [17,19]. Our finding was consistent with these previous studies. On the other hand, Nectin-4 expression was not defined as a prognostic factor in ovarian cancer [18]. Therefore, the prognostic value of Nectin-4 may be dependent on the tumor type. In this study, there were no correlations of Nectin-4 expression with various clinicopathological factors in pancreatic cancer. Data suggested that Nectin-4 might play functionally important roles in tumor progression and metastasis independently of the TNM classification. However, the underlying mechanism for the prognostic value of Nectin-4 remains unclear. Noyce et al. have reported that Nectin-4 is highly expressed in several tumor cells and plays a key role for virus entry [34]. Such a unique property of Nectin-4 may be involved in patient prognosis. Further studies will be warranted to determine the association between Nectin-4 expression and virus infection in actual pancreatic cancer.

We then investigated the potential roles of Nectin-4 in pancreatic cancer, and found several important findings. First, Nectin-4 might be involved in the proliferation of pancreatic cancer cells. This was demonstrated by the positive correlation between Nectin-4 and Ki67 expressions. Furthermore, it was also supported by the data showing that the siRNA knockdown of Nectin-4 significantly inhibits the proliferation of human pancreatic cancer cells. In fact, Takano et al. have reported a similar finding that Nectin-4 inhibition using siRNA significantly suppressed the cell proliferation in human lung cancer cells [19]. Taken together, Nectin-4 may be a critical factor contributing to the progression in certain human cancers. Furthermore, direct targeting of Nectin-4 may have therapeutic potential for cancer treatment. Therefore, it may be valuable to generate and evaluate either monoclonal antibody or small molecule compound targeting human Nectin-4.

Second, we found that there was a positive correlation of tumor Nectin-4 expression with VEGF expression and IMD in pancreatic cancer. Angiogenesis is widely accepted to play a key role in tumor growth and metastasis. And VEGF is the dominant angiogenic factor in the tumor microenvironment [35,36]. We and others have previously shown that prognostic importance and significant involvement of angiogenesis in pancreatic cancer [29,30,37]. It has recently been reported that one of nectin family, Necl-5/CD155 interacts with VEGF and induces angiogenesis in human vascular endothelial cells [22]. The members of the nectin family are known to exert a similar function and to act interactively under various conditions [12-14,38]. Therefore, we hypothesized that Nectin-4 might be associated with angiogenesis in pancreatic cancer. Although the precise underlying mechanism is still not revealed, our data suggested that Nectin-4 might be critically involved in tumor angiogenesis in pancreatic cancer. Targeting VEGF is currently a standard treatment for some human malignancies including colorectal and lung cancers. Nectin-4 blockade may enhance the effect of the inhibition of VEGF in cancer treatment. Further fundamental studies are needed to reveal the molecular mechanisms of Nectin-4 in association with angiogenesis, and to develop new therapeutic strategy.

Previous studies have demonstrated that some of the nectin family members specifically interact with the receptors on several immune cells, including T cells and NK cells, and exert immunological functions [23-27]. In addition, it has been also suggested that some members might play an important role in the tumor immunosurveillance [32,33]. We and others have reported that tumor-infiltrating CD4+, CD8+, or CD45RO+ T cells have a significant prognostic value in several human cancers, including pancreatic cancer [39-44]. Therefore, we finally evaluated the role of Nectin-4 in tumor immunity. As a result, we found no significant correlations between Nectin-4 expression and the several subsets of TILs, suggesting that Nectin-4 may have little role in modulating immune response in actual pancreatic cancer. However, careful evaluation in larger scale studies will be needed to reach a definitive conclusion.

## Conclusions

We have shown for the first time that Nectin-4 is overexpressed in human pancreatic cancer. Most importantly, tumor Nectin-4 expression has a significant prognostic value in patients with pancreatic cancer. In addition, our data suggest that Nectin-4 may contribute to tumor proliferation and angiogenesis. Further studies may be warranted to develop novel therapies targeting Nectin-4 for human malignant diseases.

## Abbreviations

UICC: International union against cancer
FBS: Fetal bovine serum
TILs: Tumor-infiltrating T lymphocytes
PCR: Polymerase chain reaction
siRNA: Small interfering RNA
VEGF: Vascular endothelial growth factor
IMD: Intratumoral microvessel density
